# Capacity of HDL to Efflux Cellular Cholesterol from Lipid-Loaded Macrophages Is Reduced in Patients with Familial Hypercholesterolemia

**DOI:** 10.3390/metabo13020197

**Published:** 2023-01-29

**Authors:** Shiva Ganjali, Susan Hosseini, Manfredi Rizzo, Anatol Kontush, Amirhossein Sahebkar

**Affiliations:** 1 Department of Medical Biotechnology and Nanotechnology, Mashhad University of Medical Sciences, Mashhad, Iran; 2 Medical Genetics Research Center, Mashhad University of Medical Sciences, Mashhad, Iran; 3 Department of Health Promotion, Mother and Child Care, Internal Medicine and Medical Specialties, School of Medicine, University of Palermo, 90133 Palermo, Italy; 4 Cardiovascular Diseases Research Unit, National Institute of Health and Medical Research (INSERM), Metabolism and Nutrition, ICAN, Sorbonne University, F-75013 Paris, France; 5 Biotechnology Research Center, Pharmaceutical Technology Institute, Mashhad University of Medical Sciences, Mashhad, Iran; 6 Applied Biomedical Research Center, Mashhad University of Medical Sciences, Mashhad, Iran; 7 Department of Biotechnology, School of Pharmacy, Mashhad University of Medical Sciences, Mashhad, Iran

**Keywords:** familial hypercholesterolemia, HDL cholesterol efflux, macrophage

## Abstract

This study aimed to evaluate the high-density lipoprotein (HDL) capacity to efflux cellular cholesterol from lipid-loaded macrophages to find a reliable and low-cost biomarker with the purpose of better evaluating the risk of premature cardiovascular (CV) events in FH patients. This case-controlled study comprised 16 homozygous (HOFH) and 18 heterozygous (HEFH) FH patients, as well as 20 healthy subjects recruited as controls. Two main subfractions of HDL (HDL2 (d = 1.063–1.125 g/mL) and HDL3 (d = 1.125–1.210 g/mL)) were isolated from the patients’ serum samples using sequential ultracentrifugation. After compositional characterization, the capacity of HDL to efflux cholesterol (CEC%) from lipid-laden macrophages was measured. The HDL2 and HDL3 subfractions showed some differences in lipid and protein composition between the studied groups. In addition, both HDL subfractions (*p* < 0.001) revealed significantly reduced CEC% in HOFH patients (HDL2: 2.5 ± 0.1 and HDL3: 3.2 ± 0.2) in comparison with the HEFH (HDL2: 3.2 ± 0.1% and HDL3: 4.1 ± 0.2%) and healthy (HDL2: 3.3 ± 0.2% and HDL3: 4.5 ± 0.3%) subjects. Additionally, multinomial logistic regression results indicated that the CEC% of both HDL2 (OR: 0.091; 95% CI: 0.018–0.452, *p* < 0.01) and HDL3 (OR: 0.118; 95% CI: 0.035–0.399, *p* < 0.01) subfractions are strongly and inversely associated with the homozygous form of FH. A decreased capacity of HDL particles to efflux cholesterol from macrophages might identify homozygous FH patients who are at elevated risk for premature CVDs. Prospective studies with a large sample size are warranted to evaluate this hypothesis.

## 1. Introduction

Familial hypercholesterolemia (FH) is a set of inherited genetic defects causing intensely increased circulating low-density lipoprotein cholesterol (LDL-C) concentrations, which can further deposit in the coronary arteries and proximal aorta and lead to elevated premature atherosclerotic cardiovascular disease (ASCVD) risk [1,2,3]. FH is diagnosed based on the identification of mutations in the genes involved in the hepatic clearance of LDL-C, including *LDL receptor (LDLR), apolipoprotein B (APOB)* and/or *proprotein convertase subtilisin/kexin type 9 (PCSK9)* [1,4].

The most frequent mutations in these genes are small nucleotide variations that are present all over the gene; while other changes, including nonsense/missense variants, altered initiation codons, frameshift variants, splicing alterations, and large deletions involving one or more exons, may also exist [5]. FH may be either heterozygous (HEFH), with the prevalence of 1 in 250 births or homozygous (HOFH), which carries biallelic pathogenic variants whose prevalence is estimated at 1:160,000 to 1:400,000 and illustrates a more severe phenotype of disease [6,7,8]. The HOFH with the LDL-C concentration of 13 mmol/L are susceptible to experiencing premature cardiovascular (CV) events at an early age [9,10].

During the lifetime, increased levels of circulating LDL-C, low high-density lipoprotein cholesterol (HDL-C) phenotype, and defective HDL functionality, may all contribute to the development of ASCVDs in these patients [11]. The main cardioprotective effect of HDL arguably involves its role in the key step of the reverse cholesterol transport (RCT) process, and the efflux of cholesterol from macrophagic foam cells [12,13,14]. As previously reported, the cholesterol efflux capacity (CEC) of HDL is impaired in FH individuals, an observation that can contribute to an increase in ASCVDs in these patients [15,16,17].

Contrary to the inheritance nature of FH, genetic testing for the detection of these patients is rarely used due to its high cost. Therefore, according to the importance of early diagnosis of homozygous forms of FH patients who poorly respond to medications and have a deficient prognosis compared to the HEFH, this study aimed to evaluate the HDL capacity to efflux cellular cholesterol from lipid-laden macrophages to find a potential biomarker with the purpose of better evaluating the risk of premature CV events in these patients.

## 2. Materials and Methods

### 2.1. Study Populations

In this case-control study, 16 patients with the homozygous form of FH (case group) from all over Iran and 18 individuals with the heterozygous form of FH (control group) among HOFH family members were recruited. The selection criteria were defined previously [18]. Briefly, FH scores for patients with suspected FH were calculated according to the Dutch Lipid Clinic Network Criteria. Then, mutations in *LDLR*, *APOB* and *PCSK9* genes were assessed by a next-generation sequencing technique and confirmed by the Sanger sequencing method. Pathogenic mutations were the ones previously identified and reported according to the ClinVar database, but for some novel mutations, their pathogenicity was predicted by the SIFT database and PolyPhen software. The HEFH patients were family members of the case group who are closely related to the HOFH patients in terms of lifestyle and genetics. In addition, 20 healthy subjects were enrolled as a control group. Written informed consent was obtained from all participants. All medical history data and blood samples were collected previously, and the patients’ serum was stored at −80 °C for further analysis. Biochemical analyses were performed using commercial kits (Pars Azmoon, Iran).

### 2.2. Isolation and Compositional Characterization of HDL Subfraction

Two main subfractions of HDL (HDL2 (d = 1.063–1.125 g/mL) and HDL3 (d = 1.125–1.210 g/mL)) were isolated from the patients’ serum samples using sequential ultracentrifugation, with different density solutions (d: 1.006, 1.21, and 1.24 g/mL), which were prepared according to the previously reported protocol [19,20,21]. Separation was achieved after a three-step ultracentrifugation followed by dialysis in PBS [22]. Thereafter, total protein (TP) (by bicinchonic acid (BCA) assay) and the main lipid components (PL, TC, TG, and FC) (by commercial kits (Diasys, France)) were quantified in the isolated HDL subfractions. Cholesteryl ester (CE) content was estimated as (TC-FC) × 1.67 [23]. The details were reported previously [22].

### 2.3. Cholesterol Efflux Assay

A HDL concentration of 30 μg protein/mL was used for the measurement of the HDL subfractions’ capacities to induce CEC in a human THP-1 monocytic cell system (ATCC) according to the previously reported protocol [23,24]. Briefly, after differentiation of the monocytes to macrophage-like cells with 50 ng/mL of phorbol 12-myristate 13-acetate (PMA), the cells’ morphology was checked under the microscope, as differentiation resulted in flat, elongated, amoeboid and adherent cells [25]. Then, the cells were loaded with [^3^H] cholesterol-labeled acetylated LDL (1 μCi/mL) for 24 h to equilibrate the cell cholesterol pools. Then, the efflux of cellular cholesterol to the HDL subfractions was evaluated after 4 h incubation of the cells in serum-free media. Finally, the CEC% was calculated as [Medium counts per minute (cpm)/(medium cpm+ cell cpm)) × 100], and by subtracting from the nonspecific CEC% that occurred in the absence of the cholesterol acceptors, and the specific CEC% was determined.

### 2.4. Statistics

All variables were normally distributed and are shown as the mean ± standard error of the mean (SE). Differences in variables were analyzed by one-way ANOVA, followed by a Tukey multiple comparison test across the 3 groups (healthy, HEFH, and HOFH), or a t-test for the independent samples between the 2 groups (HEFH and HOFH) when appropriate. The between-group differences in categorical variables were presented as percentages, and were calculated by a Chi squared analysis or by Fisher’s exact test. Pearson’s correlation coefficient was applied to evaluate the association between CEC and HDL composition. Multinomial and binary logistic regressions were also used to assess the predictive value of CEC for FH risk assessment after adjustment for age or LDL-C. SPSS software, v. 11.5 (Chicago, IL, USA) was used and *p* < 0.05 was considered as significant.

## 3. Results

### 3.1. Patients’ Characteristics

A total of 16, 18, and 20 individuals were classified as HOFH, HEFH, and healthy groups, respectively. The HOFH group included significantly younger individuals than the two other groups, although the HEFH patients were also younger than the healthy subjects (Table 1). In addition, the HOFH patients demonstrated significantly elevated levels of total cholesterol (TC), triglyceride (TG), and LDL-C in comparison with the two other groups; by contrast, the HDL-C concentration showed no significant differences between the three studied groups (Table 1).

The clinical characteristics of the FH patients are summarized in Table 2. All subjects with HOFH had xanthomas and most of them (68.8%) experienced myocardial infarction (MI). The FH score was expectedly higher in the HOFH relative to the HEFH group. Most of the HEFH patients (66.6%) did not use any drugs and 33.3% of them were on statin therapy alone, while most of the HOFH patients (75%) were on both statin and ezetimibe medication and 18.8% of them were on statin therapy (Table 2).

### 3.2. Compositional Characterization of HDL Subfractions

**Figure 1:**
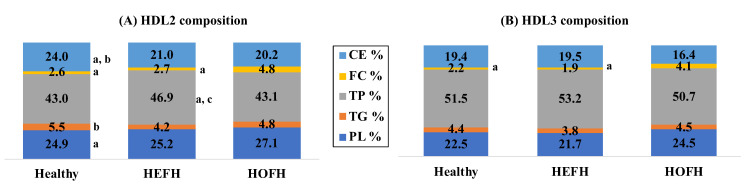
Comparison of wt% lipid and protein content of HDL subtraction between the groups; a: *p* < 0.05 vs HOFH; b: *p* < 0.05 vs HEFH; c: *p* < 0.05 vs controls. CE: cholesterol ester; FC: free cholesterol; PL: phospholipid; TG: triglyceride; TP: total protein.

As shown in Figure 1, the studied groups were different in terms of the chemical composition of HDL2 (Figure 1A) and HDL3 (Figure 1B) particles. Indeed, both the HDL2 and HDL3 particles revealed FC enrichment in the HOFH patients compared to both the HEFH (*p* < 0.001) and healthy (*p* < 0.001) subjects. In addition, a markedly reduced CE content was observed in HDL2 particles of the HEFH (*p* < 0.01) and HOFH (*p* < 0.001) patients relative to the healthy groups. By contrast, TP showed a significantly increased presence of HDL2 particles in the HEFH (*p* < 0.05) patients in comparison with the two other groups. Moreover, the healthy subjects showed a significantly elevated TG (*p* < 0.01) and reduced PL (*p* < 0.05) content in the HDL2 particles as compared with the HEFH and HOFH, respectively (Figure 1).

The HDL2 subfraction showed a significantly decreased PL/TP ratio in the HEFH compared with the HOFH (*p* < 0.05) group, while the TC/TP ratio was elevated in the HOFH (*p* < 0.05) and healthy (*p* < 0.05) groups as compared with the HEFH group. Finally, the HDL2/HDL3 ratio was also decreased in the HOFH (*p* < 0.01) patients compared to the two other groups (Table 1).

### 3.3. The Percentage of Cholesterol Efflux Capacity of HDL Subfractions

Both HDLs (*p* < 0.001) revealed significantly reduced CEC% in the HOFH patients relative to the HEFH and healthy subjects (Table 3). However, a significantly reduced CEC/HDL-C ratio was only found for HDL3 (*p* < 0.05) in the HOFH patients relative to healthy individuals (Table 3). In addition, females displayed the markedly highest HDL2 CEC% (*p* < 0.01) compared with the males in the HOFH patients. By contrast, age did not affect the CEC% in any of the studied groups (data not shown).

**Figure 2:**
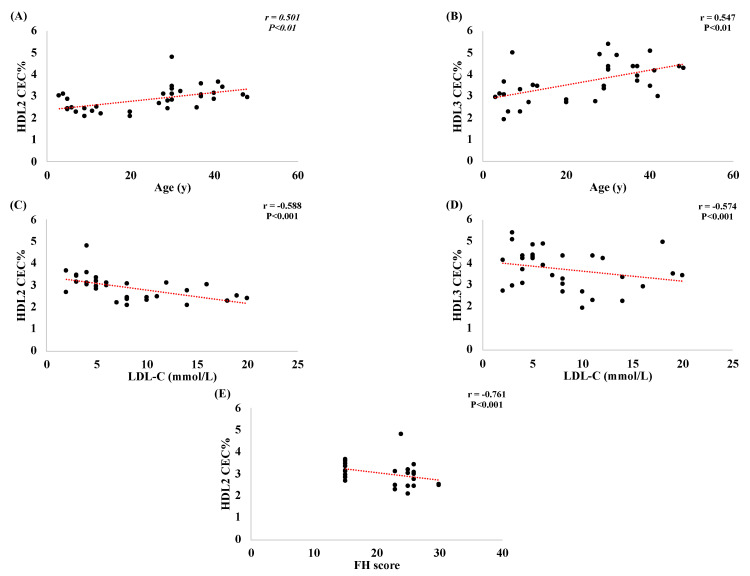
Correlations between the CEC% of HDL subfractions and age (**A**,**B**), LDL-C (**C**,**D**), and FH score (**E**) in all FH patients, combined.

In addition, there was no correlation between the CEC% of both the HDL2 and HDL3 and the age and lipid profile in all studied groups (data not shown). Albeit, when all of the HEFH and HOFH patients were analyzed together (as FH patients), there was a strong positive relationship between both HDL2 CEC% (Figure 2A) and HDL3 CEC% (Figure 2B) and age. Moreover, LDL-C levels were negatively correlated with HDL2 CEC% (Figure 2C) and HDL3 CEC% (Figure 2D). Finally, the FH score showed a strong negative correlation with HDL2 CEC% (Figure 2E).

Results of the multinomial logistic regression revealed that the HDL2 CEC% and HDL3 CEC% subfractions, as well as the HDL2 CEC/HDL-C and HDL3 CEC/HDL-C ratios, were strongly and inversely associated with the homozygous form of FH (Table 4). However, after an adjustment for age (Table 4) and LDL-C (data not shown), these associations disappeared. In addition, the results of the binary logistic regression demonstrated strong and inverse relationships between HDL2 CEC% and HDL3 CEC% and HOFH (Table 5). After an adjustment for age (Table 5) and LDL-C (data not shown) as a confounder, these associations were weakened.

## 4. Discussion

HDL-C is a traditional CVD risk marker that reflects cholesterol content [26,27]. However, HDL is a complex particle containing various enzymes, lipids, and proteins that can influence its cardioprotective properties. Therefore, the assessment of HDL composition and function has been suggested to be used for evaluating CVD risk, especially in FH patients who are at high risk for CVD [11,28].

Hypercholesterolemia promotes compositional alterations in HDL particles, thereby impairing their ability to induce cholesterol efflux from macrophages [29]. In this case-controlled study, the HDL2 subfraction showed TG enrichment and PL and CE depletion in both the HEFH and HOFH patients when compared to healthy subjects. Moreover, both the HDL2 and HDL3 subfractions revealed FC enrichment in the HOFH patients compared to the other groups. However, markedly elevated TP content was observed in the HDL2 subfractions of HEFH patients, which was accompanied by reduced PL content resulting in the diminished PL/TP ratio in these patients when compared to the HOFH group. However, the HDL2 TC/TP ratio was elevated in HOFH patients compared to the HEFH group, which could reflect increased plasma cholesterol levels in HOFH. Reduced PL content of the HDL3 particles was previously reported in FH patients relative to healthy controls [30]. Moreover, TG-rich HDL3 particles were present in FH patients with premature coronary heart disease [31]. Moreover, in the present study, the HDL2/HDL3 ratio was decreased in the HOFH patients compared to the other groups, potentially reflecting the decreased HDL2 total mass in HOFH. In addition, our group previously reported the excessive oxidative burden in HOFH patients; therefore, increased FC content in both the HDL2 and HDL3 particles of HOFH patients might be an important source of substrates to be oxidized by different oxidative agents in these patients leading to HDL dysfunction. It has been reported that HDL oxidation weakens the lipoprotein’s capability to clear cells from cholesterol and this might explain the pro-atherogenic behavior of HDL particles [32].

The efflux of cholesterol from lipid-laden macrophages is a key cardioprotective mechanism against excess cholesterol uptake, so it is an important process in preventing the transformation of lipid-laden cells into foam cells and, subsequently, atherosclerotic plaque [33]. Numerous studies have reported an abnormal CEC in FH patients [15,34,35,36,37]. In the present study, impaired CEC was also found in both the HDL2 and HDL3 subfractions from HOFH patients relative to the HEFH and healthy groups. Several reports documented a decreased capacity of HDL from FH patients to efflux cholesterol from cultured macrophages [15,17,34]. In addition, plasma from HEFH patients with CVD displayed a reduced capacity to efflux cholesterol compared to HEFH patients without CVD, possibly reflecting differences in HDL composition [38]. Consistent with this hypothesis, the HDL2 CEC% showed, respectively, a positive and negative correlation with TP and PL content of HDL2 from all FH patients in the present study (data not shown). Since our HOFH patients were younger subjects, we assessed the relationship between the CEC% of both the HDL2 and HDL3 and age in our group and found no significant correlation. However, when all of the HEFH and HOFH patients were combined, a correlational analysis revealed a strong positive relationship between both HDL2 CEC% and HDL3 CEC% and age. Moreover, LDL-C levels were negatively correlated with the HDL2 CEC% and HDL3 CEC% in all FH patients combined.

It was reported that altered HDL remodeling and composition was associated with low HDL-C levels and dysfunctional RCT [39]. Despite an impaired CEC% in FH patients, HDL-C showed no statistical differences between the groups in the present study. Therefore, we normalized the CEC% of both HDL subfractions to HDL-C levels, and only observed a significantly reduced HDL3 CEC/HDL-C ratio in the HOFH patients vs. healthy individuals, potentially reflecting a markedly diminished HDL3 CEC% in HOFH.

Furthermore, it was reported that male gender altered the HDL size and function [40]. In a subgroup analysis, the CEC% of the HDL subfraction was, therefore, compared between males and females in each group. The results indicated that gender affected the CEC%, so that females displayed a markedly elevated HDL2 CEC% than males in the HOFH patients.

Moreover, the HDL CEC% showed an inverse and independent association with the presence of ASCVD (OR: 0.95; 95% CI: 0.90–0.99) in earlier studies [15]. Specifically, the impaired HDL2 CEC found in the HOFH and HEFH patients showed an inverse association with the development of atherosclerosis [41]. This association was confirmed when a reduced CEC% was observed in HDL subfractions of HEFH patients relative to normolipidemic individuals, revealing a relationship with CVD risk [42]. The results of multinomial logistic regression showed that both the HDL2 CEC% and HDL3 CEC%, as well as the HDL2 CEC/HDL-C and HDL3 CEC/HDL-C ratios, were strongly and inversely associated with the homozygous form of FH. However, after an adjustment for confounders, including age and LDL-C, these associations disappeared. In addition, a strong and inverse relationship between HDL2 CEC% and HDL3 CEC% and HOFH was found in the binary logistic regression analysis. Similarly, after an adjustment for age and LDL-C as confounders, the association was weakened, reflecting the strong effects of the two confounders on CEC%.

In conclusion, our data suggest that the reduced capacity of HDL particles to efflux cellular cholesterol from macrophages might identify homozygous FH patients who are at high risk for premature ASCVDs. However, the small sample size of our study was one of the most important limitations in this study, which does not grant a definite conclusion. Nevertheless, including the HEFH group as a valid control group, the subjects of which were closely related to the HOFH patients in terms of lifestyle and genetics, is one of the strengths of this pilot case-controlled study. A lack of a detailed assessment of the comorbidities in the studied groups is another potential limitation. According to our results, impaired CEC from cholesterol-laden macrophages could be considered a potential marker for HOFH patients, and promoting RCT and cholesterol efflux processes can be a potentially promising strategy for reducing CV events in FH patients. This has a particular clinical relevance, since a more tailored approach with statins, PCSK9 targeting drugs and other lipid-lowering agents [39], is needed in order to improve the CV outcome of FH patients. Prospective studies with a large sample size are warranted to evaluate this hypothesis.

## Figures and Tables

**Table 1:** Baseline characteristics of the subjects.

Variables		Familial Hypercholesterolemia (N = 34)		Healthy (N = 20)	*p*-Value
		HEFH (N = 18)	HOFH (N = 16)		
Sex	Male	10 (55.6%)	5 (31.3%)	10 (50.0%)	0.361
	Female	8 (44.4%)	11 (68.8%)	10 (50.0%)	
Age (y)		33.61 ± 2.3 ^a^	14.0 ± 2.7	48.5 ± 1.9 ^a,b^	<0.001
TC (mmol/L)		6.4 ± 0.5 ^a^	16.1 ± 1.2	4.9 ± 0.2 ^a^	<0.001
TG (mmol/L)		1.2 ± 0.1 ^a^	2.3 ± 0.4	1.4 ± 0.1 ^a^	<0.01
HDL-C (mmol/L)		1.6 ± 0.3	1.6 ± 0.2	1.3 ± 0.1	0.376
LDL-C (mmol/L)		4.3 ± 0.4 ^a^	11.9 ± 1.1	2.9 ± 0.1 ^a^	<0.001
HDL2	PL/TP	0.5 ± 0.0 ^a^	0.6 ± 0.0	0.6 ± 0.0	<0.05
	TC/TP	0.3 ± 0.0	0.4 ± 0.0 ^b^	0.4 ± 0.0 ^b^	<0.01
HDL3	PL/TP	0.4 ± 0.0	0.5 ± 0.0	0.4 ± 0.0	0.080
	TC/TP	0.3 ± 0.0	0.3 ± 0.0	0.3 ± 0.0	0.734
HDL2/HDL3		2.6 ± 0.1 ^a^	1.6 ± 0.2	2.4 ± 0.2 ^a^	<0.001

Data are shown as the mean ± SE; ^a^: Significant in comparison with HOFH group; ^b^: Significant in comparison with HEFH group. HDL-C: High-density lipoprotein cholesterol; HEFH: Heterozygous familial hypercholesterolemia; HOFH: Homozygous familial hypercholesterolemia; LDL-C: Low-density lipoprotein cholesterol; mmol/L: Millimoles per liter; TC: Total cholesterol; TG: Triglyceride; y: Year.

**Table 2:** Clinical characteristics of the FH patients.

Variables		Familial Hypercholesterolemia (N = 34)		*p*-Value
		HEFH (N = 18)	HOFH (N = 16)	
FH score		15.0 ± 0.0	25.6 ± 0.5	<0.001
Number of patients with xanthoma symptoms		0.0%	100.0%	<0.001
Number of patients with MI history		0%	68.8%	<0.001
Mutation (%)	Previously reported	84.6%	84.6%	1.000
	Novel	15.4%	15.4%	
Mutation type (%)	Missense	44.4%	50.0%	1.000
	Truncated	27.8%	25.0%	
	Single nucleotide variant	16.7%	12.5%	
	Single nucleotide polymorphism	11.1%	6.3%	
	Missense, truncated	0.0%	6.3%	
Position of LDLR mutation (%)	Exon	72.7%	84.6%	0.630
	Intron	27.3%	12.5%	
Drugs consumption (%)	No drug	66.7%	6.2%	<0.01
	Only statin	33.3%	18.8%	
	Statin + ezetimibe	0%	75.0%	

FH: Familial hypercholesterolemia; LDLR: Low-density lipoprotein receptor; MI: Myocardial infarction.

**Table 3:** CEC% of HDL from FH patients and healthy controls.

Variables	Familial Hypercholesterolemia (N = 34)		Healthy (N = 20)	*p*-Value
	HEFH (N = 18)	HOFH (N = 16)		
HDL2 CEC (%)	3.2 ± 0.1 ^a^	2.5 ± 0.1	3.3 ± 0.2 ^a^	<0.001
HDL3 CEC (%)	4.1 ± 0.2 ^a^	3.2 ± 0.2	4.5 ± 0.3 ^a^	<0.001
HDL2 CEC/HDL-C	2.3 ± 0.2	1.9 ± 0.3	2.6 ± 0.2	0.126
HDL3 CEC/HDL-C	2.9 ± 0.3	2.5 ± 0.3	3.6 ± 0.3 ^a^	<0.05

Data are shown as the mean ± SE; ^a^: Significant in comparison with HOFH group. HDL-C: High-density lipoprotein cholesterol; HEFH: Heterozygous familial hypercholesterolemia; HOFH: Homozygous familial hypercholesterolemia; CEC: Cholesterol efflux capacity.

**Table 4:** Multinomial logistic regression for CEC in relation to FH status (Reference: healthy controls).

Variables	HOFH				HEFH			
	Unadjusted		Adjusted ^#^		Unadjusted		Adjusted ^#^	
	OR (95% CI)	*p* Value	OR (95% CI)	*p* Value	OR (95% CI)	*p* Value	OR (95% CI)	*p* Value
HDL2 CEC (%)	0.091 (0.018–0.452)	<0.01	0.145 (0.018–1.175)	0.071	0.864 (0.330–2.258)	0.765	1.631 (0.386–6.887)	0.505
HDL3 CEC (%)	0.118 (0.035–0.399)	<0.01	0.306 (0.062–1.498)	0.144	0.681 (0.345–1.343)	0.268	0.763 (0.295–1.972)	0.577
HDL2 CEC/HDL-C	0.439 (0.194–0.991)	<0.05	1.149 (0.183–7.209)	0.882	0.721 (0.321–1.623)	0.430	1.187 (0.303–4.652)	0.805
HDL3 CEC/HDL-C	0.452 (0.240–0.852)	<0.05	0.860 (0.196–3.777)	0.841	0.680 (0.240–1.250)	0.214	0.856 (0.318–2.301)	0.758

#: Adjusted for age. CI: Confidence interval; HEFH: Heterozygous familial hypercholesterolemia; HOFH: Homozygous familial hypercholesterolemia; OR: odds ratio.

**Table 5:** Binary logistic regression for CEC in relation to HOFH (Reference: HEFH).

Variables	Unadjusted		Adjusted ^#^	
	OR (95% CI)	*p* Value	OR (95% CI)	*p* Value
HDL2 CEC (%)	0.004 (0.000–0.123)	<0.01	0.010 (0.000–0.533)	<0.05
HDL3 CEC (%)	0.181 (0.055–0.595)	<0.01	0.385 (0.101–1.471)	0.163
HDL2 CEC/HDL-C	0.669 (0.300–1.490)	0.325	1.005 (0.254–3.966)	0.995
HDL3 CEC/HDL-C	0.708 (0.383–1.311)	0.272	1.013(0.317–3.241)	0.982

#: Adjusted for age. CI: Confidence interval; HEFH: Heterozygous familial hypercholesterolemia; HOFH: Homozygous familial hypercholesterolemia; OR: odds ratio.

## Data Availability

Data is contained within the article.
